# Brain retraction injury after elective aneurysm clipping: a retrospective single-center cohort study

**DOI:** 10.1007/s00701-022-05131-y

**Published:** 2022-02-02

**Authors:** B. Konya, J. W. Dankbaar, A. van der Zwan

**Affiliations:** 1 Department of Neurosurgery, UMC Utrecht, Utrecht, the Netherlands; 2 Department of Radiology, UMC Utrecht, Utrecht, the Netherlands

**Keywords:** Vascular neurosurgery, Brain retraction injury (BRI), Aneurysm, Radiology, Retractors

## Abstract

**Background:**

BRI is estimated to occur in 10% of skull-base surgery and 5% of aneurysm surgery. These estimates are based on a few studies with unclear methodology. The purpose of this study is to assess the rate of BRI occurrence, its risk factors, and the association between BRI and postoperative focal neurological deficit in patients that underwent elective aneurysm surgery in a single institution.

**Methods:**

All patients that underwent elective aneurysm surgery in a single tertiary center in the Netherlands were included. BRI was defined as cortical hypodensities in the surgical trajectory not matching areas of large arterial infarction. Risk ratios were calculated between BRI and (a) the use of temporary parent artery occlusion during clipping, (b) anterior communicating artery (ACom), and (c) middle cerebral artery (MCA) location of the aneurysm, (d) presence of mentioned CVA risk factors, (e) the clipping of > 1 aneurysm during the same procedure, and (f) new focal neurological deficit. Statistical analysis further included *t*-tests and binary logistical regression analysis on the correlation between age and BRI.

**Results:**

BRI was identified postoperatively in 42 of the 94 patients included in this study. A new focal neurological deficit was found in 7 patients in the BRI group. A total of 5 patients had persisting symptoms at 3-month follow-up, of which 2 were caused by BRI. Increasing age is a risk factor for developing BRI.

**Conclusions:**

The high rate of BRI and significant risk of new postoperative focal neurological deficit in our patients should be considered when counseling patients for elective aneurysm surgery.

## Introduction

Brain retractors have been an indispensable instrument for neurosurgeons since the nineteenth century when medical advancements allowed for surgeons to operate deep intracranially for the first time [2]. Although the use of retractors is a daily practice for neurosurgeons, it is not known how much tissue damage is caused by the retractors. Several studies have implied an association between excessive brain retraction and postoperative brain retraction injury (BRI) [1, 5, 7, 8]. BRI may be caused by long or firm usage of brain retractors which can lead to direct injury or vessel compression [10]. Subsequently, brain edema, ischemia, and infarction of the brain parenchyma may occur [1].

The use of new retracting instruments has been proposed to decrease this risk, as well as the use of certain retraction techniques [3]. Intermittent retraction may be beneficial as animal studies have shown the occurrence of irreversible brain damage to be related to both retraction pressure and duration [7]. Application of retraction pressure above 30 mmHg or a local brain perfusion pressure under 10 mmHg for 6 to 8 min may lead to irreversible damage [7]. A study in dogs showed that continuous retraction with 22 mmHg pressure for 60 min led to brain lesions in 50% of the cases, while the use of intermittent retraction (6 repetitions of 10 min of retraction followed by 5 min of rest) only led to lesions at 30 mmHg or higher [9]. Maximum retraction force and intermittence times in humans are unknown and may be individually determined by collaterals and intraoperative perfusion conditions.

BRI is estimated to occur in 10% of skull-base surgery and 5% of aneurysm surgery [1]. These estimates of BRI occurrence are based on a few studies with small heterogeneous study populations and unclear methodology of diagnosing BRI [5, 7, 8]. The process of finding solutions to decrease patients’ risk for BRI is complicated by uncertainties regarding its occurrence and its clinical consequence for the patient [1, 5, 7, 8].

The purpose of this study is to retrospectively assess the rate of BRI occurrence, its risk factors, and the association between BRI and postoperative focal neurological deficit in patients that underwent elective aneurysm surgery at our institution.

## Methods and materials

We retrospectively identified all patients that underwent elective aneurysm surgery in the University Medical Center in Utrecht, the Netherlands, between January 1, 2009 and December 31, 2019 and met the following inclusion criteria: (a) age of 18 years and older, (b) clipping of an unruptured saccular aneurysm, including giant aneurysms, (c) postoperative CT angiography (CTA) and non-contrast CT (NCCT) of the brain, routinely performed in our institution for postoperative evaluation of aneurysm occlusion, available within 90 days after surgery. Excluded were those patients in which other surgery techniques were used for treating the asymptomatic aneurysm (wrapping or bypass), patients with a subarachnoid hemorrhage (SAH) from another aneurysm within 30 days before surgery, and incomplete data. The study was evaluated by the Medical Ethics Committee of the University Medical Center in Utrecht, and the need for informed consent was waived.

Brain retraction was performed throughout surgery using intermittent retraction as is standardized in our department. Traditional Leyla retractor blades (Braun) were used, fixed to a frame on the operating table (Mayfield) or skull (Sugita). Retractor blades were repositioned every few minutes to protect the brain from retractor-induced damage. In addition, excessive retraction forces were avoided by suctioning enough cerebrospinal fluid throughout the surgery. This technique was used by all neurosurgeons who operated on patients participating in this study.

The following patient characteristics were collected: age, sex, presence of CVA risk factors (diabetes or hypertension or smoking or hypercholesterolemia); imaging (postoperative CTA and NCCT of the brain during the first 90 days after surgery); surgery data (duration of surgery, use of temporal clipping, location of operated aneurysm, number of aneurysms clipped during each surgical procedure); the occurrence of new postoperative focal neurological deficit in the first 3 days after surgery; persisting new postoperative focal neurological deficit at 3 months. Postoperative CT imaging was assessed for BRI by two observers blinded for patient outcome, one neuroradiologist (J.D.) and one neurosurgeon (A.Z). BRI was defined as cortical hypodensities adjacent to the surgical trajectory not matching areas of large arterial infarction (for example, due to occlusion of the recurrent artery of Heubner). For all discrepancies, consensus was reached.

Statistical analyses were performed using IBM SPSS Statistics 27.0 (IBM Corporation, Armonk, NY). The occurrence rate of postoperative BRI on CT imaging was assessed in the descriptive analysis. In the main analysis, the means of continuous variables age and surgery duration were compared between the groups with and without BRI using independent *t*-tests. Statistical significance was set at a *p*-value < 0.05. Afterward, risk ratios were calculated between BRI and the following categorical variables: (a) the use of temporary parent artery occlusion during clipping, (b) anterior communicating artery (ACom), and (c) middle cerebral artery (MCA) location of the aneurysm, (d) presence of mentioned CVA risk factors, (e) the clipping of > 1 aneurysm during the same procedure, and (f) new focal neurological deficit. Finally, binary logistic regression was used to calculate a correlation between age and the odds of developing postoperative BRI.

## Results

**Fig. 1:**
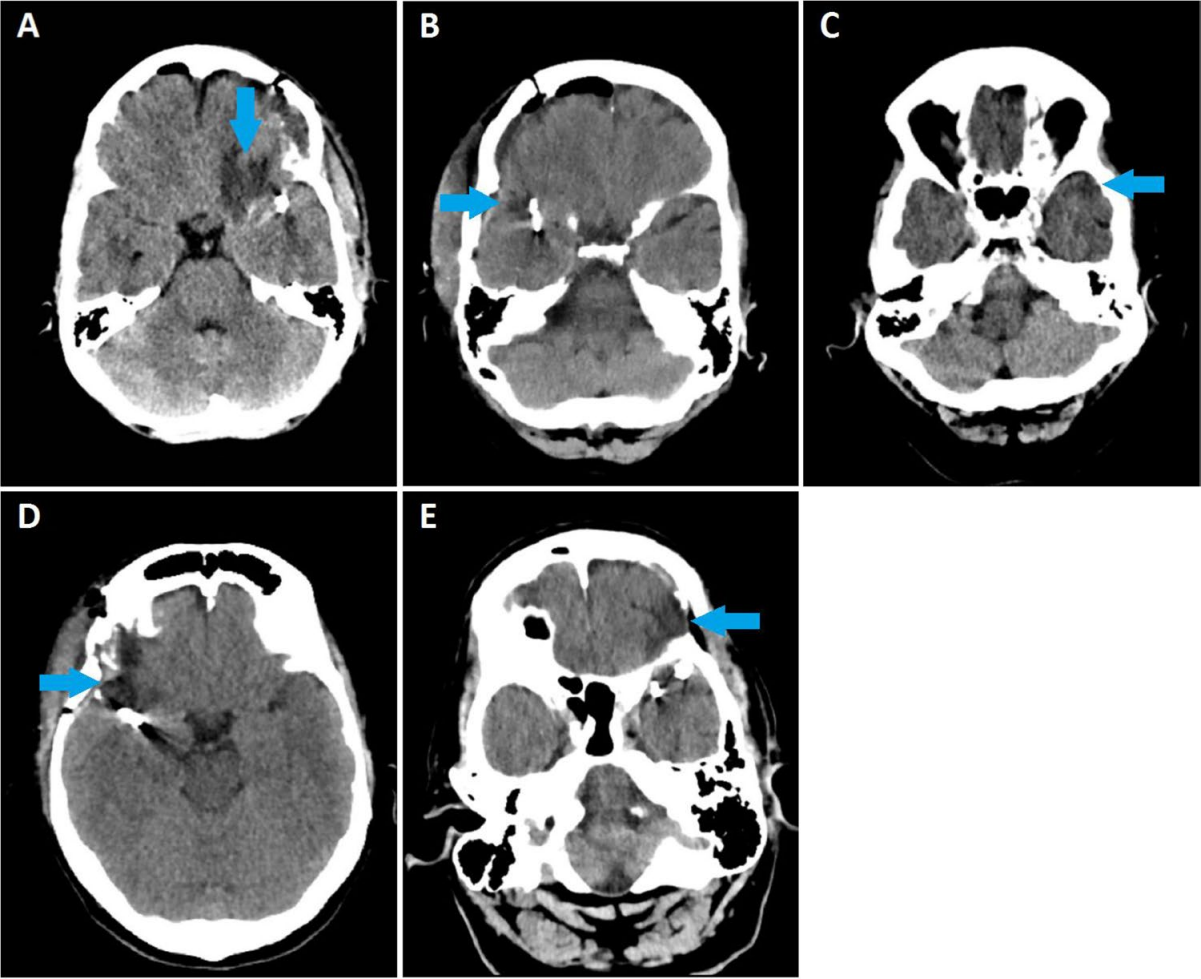
A–E panels of 5 CT scans (**A**–**E**). Arrows point to BRI. **A** CT scan of a severe case of BRI after clipping of a large MCA aneurysm. **B–E** Examples of CT scans with minor BRI

A total of 114 operated aneurysms in 94 patients were included in this study. BRI was identified postoperatively in 42 of the 94 patients (Fig. 1). The median time of brain imaging after surgery was 4 ± 25 days, range (0–79). The average age of patients was 54.8 ± 10.1, range (0–79) (Table 1). Overall, 66 of 94 (70%) patients were female. Postoperative new focal neurological deficit was established in 7 patients with BRI. Four out of 7 patients had BRI localizations correlating to their clinical symptoms (Table 2). At 3-month follow-up, 5 patients had persisting deficit symptoms, of which 2 were correlated. None of the patients without BRI had a postoperative new focal neurological deficit.

**Table 1:** Baseline

Patient characteristics	Patients without BRI (52)	Patients with BRI (42)	Total (94)	*P*-value
Sex				
Female	37	29	66	
Male	15	13	28	
CVA risk factors	44	33	94	
Age, mean	51.3 ± 10.4	59.0 ± 8.1	54.8 ± 10.1	< **0.001**
Surgery duration, mean (minutes)	183 ± 76	193 ± 58	188 ± 68	0.463
Surgery duration MCA aneurysm, mean (minutes)	181 ± 76	195 ± 62	187 ± 69	0.425
Surgery duration Acom aneurysm, mean (minutes)	197 ± 63	188 ± 51	192 ± 55	0.783
ACom clipped	6	7	13	
MCA clipped	30	27	57	
Other aneurysm locations clipped	16	8	24	
Multiple aneurysms clipped	8	8	16	
Postoperative new focal NEU deficit	0	7	7	
Persisting postoperative focal NEU deficit at 3-month follow-up	-	5	5

**Table 2:** Overview of patients with postoperative focal neurological deficit

Patient	Aneurysm location	Description of focal neurological deficit	Deficit at 3-month follow-up	Correlation between BRI and focal neurological deficit?
1	Right MCA and PCOM	Central facial paresis left and paresis of the left arm	Persisting	Yes
2	Left MCA	Dysfasia, hemiparesis right including a facial paresis and neglect of the right side	Persisting	No
3	Left A1-segment	Dysfasia, hemiparesis right including a facial paresis and neglect of the right side	Recovered	Yes
4	Right MCA and ACOM	Paresis of the left arm and leg	Persisting	No
5	Left MCA	Hemiparesis right including a facial paresis	Persisting	No
6	Right MCA	Hemiparesis left including a facial paresis and a neglect of the right side	Recovered	Yes
7	Left A1-A2	Afasia and paresis of the right arm and leg	Persisting	Yes

### T-test and risk analysis

Mean age was significantly different between the patients with (59.0 years) and without BRI (51.3 years) (*p* < 0.001). Mean surgery duration was not significantly different between patients with (183 min) and without BRI (193 min) (*p* = 0.463). Mean surgery duration in subgroups (MCA aneurysm surgery only or ACom aneurysm surgery only) was also not significantly different (*p* = 0.425 and *p* = 0.783, respectively).

Relative risks (RR) on postoperative BRI were calculated for factors mentioned before. The RR of temporary parent artery occlusion on BRI is 1.480 (95% CI, 0.834–2.629), RR of age > 60 on BRI is 1.840 (95% CI, 1.263–2.681), RR of CVA risk factors on BRI is 0.718 (95% CI, 0.303–1.699), RR of ACom localization on BRI is 1.062 (95% CI, 0.898–1.255), RR of MCA localization on BRI is 1.185 (95% CI, 0.708–1.983), and the RR of multiple aneurysms clipped on BRI is 1.044 (95% CI, 0.524–1.721). The absolute risk of BRI on developing focal neurological deficit is 0.167.

### Binary logistic regression analysis

We calculated the regression coefficient of the independent continuous variable age on our dependent binary variable BRI using logistic regression analysis. We found a *B* value of 0.482 (*p* = 0.001), and per 5-year increase in age, the odds for developing BRI increases with factor 1.620.

## Discussion

The aim of this study was to assess the rate of BRI, its possible risk factors, and the association between BRI and focal neurological deficit. BRI was found on postoperative CT imaging in 46% of the cases that were clipped between 2009 and 2019. A total of 16% of the patients with BRI showed a new focal neurological deficit, while none showed a new deficit in the group without BRI. Of the patients with a new focal neurological deficit, the deficit persisted in 5 of the 7 patients 3 months postoperatively. However, only in two patients, a relationship was found between the hypodensities we assessed as BRI and the postoperative focal neurological deficit. For the other three patients, the focal neurological deficit could not be correlated to the BRI hypodensities and was most likely caused by other complicating factors. Increasing age is a risk factor for developing BRI.

Prolonged artery occlusion is a known risk factor for postoperative ischemia, [4] but in our study, we found no association with BRI and the use of temporary artery occlusion. This is not surprising considering our definition of BRI: cortical hypodensities in the surgical trajectory not matching areas of large arterial infarction. Because of our definition, hypodensities caused by temporal artery occlusion that most likely do manifest in areas of large arterial infarction were not classified as BRI. This supports the idea of localized small infarctions following local compression of superficial cortical arterioles and capillaries by retractor pressure. Also, CVA risk factors and surgery with multiple aneurysms clipped were not significantly associated with BRI. The average duration of surgery was not significantly different between the cases with and without BRI.

Although it could be hypothesized that older patients with atrophic brains may be protected from BRI compared to the younger patient, we found age to be a risk factor for BRI. Brain retraction may be more detrimental in an older brain due to a higher sensitivity to oxygen deprivation. Mice models, for example, show an association between age and decreased oxygen availability and capillary loss in the brain tissue [6].

To our knowledge, no previous study has investigated the rate of postoperative cortical hypodensities in the surgical area. Previous research suggested lower rates (5–10%) of brain retraction injury after neurosurgical procedures, without providing a clear definition of what was perceived as BRI. The results in this study suggest a much higher rate (46%) of BRI. By choosing the electively clipped population with relatively healthy brains, we purposefully attempted to eliminate factors that could contribute to postoperative cortical damage (i.e., vasospasms after SAB’s, affected brain tissue due to tumors, or other causes and predispositions for postoperative brain damage). In contrast, previous studies combined both patients with ruptured aneurysms and brain tumors in their study population [5, 7, 8], possibly influencing how the origin of postoperative cortical damage was assessed. Thus, a possible explanation for the difference in BRI rates may be due to a difference in study populations and the definition of BRI.

Our study did have limitations. We were unable to retrospectively gather data regarding the duration of the temporary artery occlusion as well as the duration of brain retraction. For the duration of brain retraction, we attempted to use surgery duration as a proxy in this study. Both a longer duration of temporary parent artery occlusion and brain retraction may be associated with BRI and should be included and possibly adjusted for in future research. Additionally, the effects of BRI on cognitive functioning or the long-term clinical consequences should also be assessed in future research in larger study populations.

Selection bias may also play a role in our results. This study was conducted in a tertiary medical center in the Netherlands with expertise in aneurysm surgery. In the present era of endovascular treatment of intracranial aneurysms, the neurosurgeon has been confronted with only the more complex and larger (giant) aneurysms. This means that the patient population and results should be interpreted accordingly.

Finally, in this retrospective study, we obtained the presence of persisting focal neurological deficit at 3-month follow-up from physicians’ notes. Therefore, it is possible that some form of underrepresentation of focal neurological deficit may be present as minor deficits could in theory be overlooked.

## Conclusion

The occurrence rate of BRI in this study was found to be 46%. This is higher than suggested in previous research. Patients with BRI had an absolute risk of a new focal neurological deficit of 0.167. Only 2 out of 94 patients showed persisting postoperative neurological deficit at 3-month follow-up that was most likely due to BRI. Age is associated with an increased risk for BRI. However, temporary artery occlusion, CVA risk factors, ACom or MCA aneurysm location, or surgery with multiple aneurysms clipped were not associated with an increased risk for BRI in this study. Surgery duration was not significantly different between patients with and without BRI. The high rate of BRI and significant risk of new postoperative focal neurological deficit in our patients should be considered when counseling patients for elective aneurysm surgery.

## Abbreviations

BRI: Brain retraction injury
CTA: CT angiography
NCCT: Non-contrast CT
SAH: Subarachnoid hemorrhage
